# Classification of the Cutting Surface Topography Using a Set of Uncorrelated Parameters with High Discriminative Ability

**DOI:** 10.3390/ma18133131

**Published:** 2025-07-02

**Authors:** Rafal Rozanski, Elzbieta Kawecka, Andrzej Perec

**Affiliations:** Faculty of Technology, Jacob of Paradies University, 66-400 Gorzów Wielkopolski, Poland; rrozanski@ajp.edu.pl (R.R.); ekawecka@ajp.edu.pl (E.K.)

**Keywords:** roughness, cutting, geometric structure of the surface, classification ability of parameters

## Abstract

The paper proposes a new coefficient assessing the classification ability of parameters. In contrast to previously used indices, it does not require data normalization, examines the correlation between parameters with the highest classification ability, and determines, based on this, a complementary set that enables effective differentiation of surfaces that differ significantly. The empirical part is based on the values of 83 parameters that characterize the stereometric features of 22 surfaces created through different machining processes.

## 1. Introduction

The important technological issue in manufacturing is the evaluation of stereometric features of technical surfaces. The structure of the machined surface is directly related to the technology used to obtain it. Cutting is characterized by a relatively high structure roughness with high processing efficiency. Other technologies, such as grinding [1,2,3], micro-cutting [4], or plastic deformation [5,6,7], yield a low roughness of the obtained surface. On the other hand, other erosion techniques like AWJ machining [8,9] allow for achieving a relatively high efficiency of the machined surface while maintaining very high quality. The best surface structure after machining, especially super-hard materials, is achieved using EDM [10,11]. Machined material properties also influence the desired surface quality achievements after the treatment. In the case of hard [12,13] or difficult-to-machine materials [14,15], a different stereometry of the machined surface should be expected than, for example, for relatively soft bearing materials [16] or thin to machining layers [17].

Many methods [18] and parameters are used for surface quality evaluation. Over three hundred parameters are used. None allow for an unambiguous distinction between significantly different surfaces. Many surfaces that differ significantly but are characterized by similar parameter values can be used to assess the surface condition [19].

Several parameters should be used to differentiate surfaces. However, there should not be too many of them because this may limit the possibility of comparing surfaces that are better in one parameter term, but worse in other terms. Hence, an important quality element of assessing technical products is the selection of a complementary set of parameters that ensures ease of interpretation of assessments for specific applications and surfaces and the ability to distinguish significantly different surfaces more effectively. This feature was named the parameter classification ability of the parameter [20].

## 2. Theoretics

### 2.1. Classification Ability

**Figure 1:**
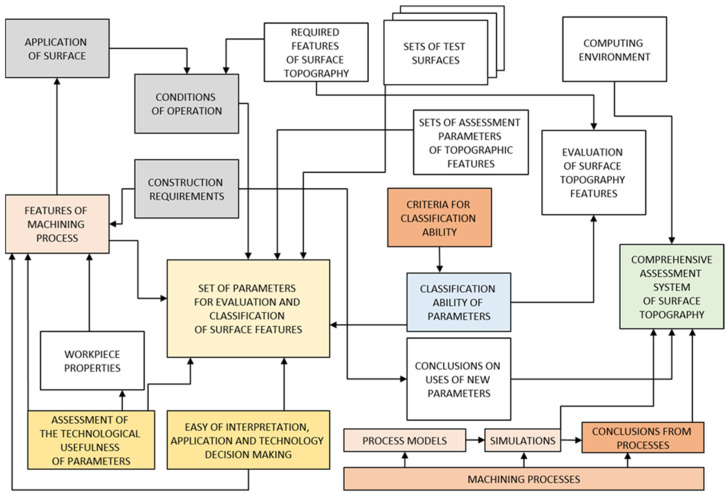
Schematic of the methodology for selecting a set of parameters with high classification ability (own study based on [28]).

The classification issues are described extensively in the literature on general statistical tools [21,22,23,24,25] as well as on technical products or directly related to the surface structure [26,27]. The chart presents the degree of process complexity in the set parameter selection, characterized by a high classification ability. It is easy to interpret and useful in the parameter selection frame and the machining process conditions, as presented in Figure 1.

The parameters discussed in the literature usually have different value ranges. In comparison, the normalized value was often used. Some authors [20,29,30] used classical normalization based on the minimum and maximum values of the parameter value range, as well as normalization that takes into account the basic statistics related to the distribution of parameter values, such as the average parameter value. In the abovementioned works, normalization was also proposed using the limit values derived from the current state of knowledge for each analyzed parameter.

The classification ability of a parameter increases when the equalization of differences between its successive values for different surfaces increases, i.e., when the probability distribution of differences in parameter values tends to a uniform distribution. The highest theoretical classification ability will be achieved when the differences between successive values of the standardized parameter $P_{j}$ for individual surfaces are equal, i.e., for each i=1,…,n−1 we have:

(1) $$∆P_{j_{i}}=P_{j_{i+1}}-P_{j_{i}N}=\frac{1}{n-1},$$

where *n* is the number of tested surfaces.

In connection with the above, the variance of the increments of its value can indicate the parameter classification ability. Let us therefore assume the following definition

**Definition** **1.** *The parameter* $P_{j}$ *has the highest V-classification ability when the condition is met*

(2) $$Var\left(∆P_{j_{i}}\right)=\underset{k}{\min}Var\left(∆P_{k_{i}}\right),$$

*where* $Var\left(∆P_{k_{i}}\right)$ *means the variance of the parameter increments* $P_{k}$.

The classification ability of a parameter will increase when the variance of its increments decreases to 0. The disadvantage of using variance as an indicator of classification ability is its low resistance to the appearance of outliers.

Regarding the optimality criteria adopted in statistics concerning the selected criteria [22], the following criteria of classification ability were defined in [29,31].

**Definition** **2.** *Parameter* $P_{j}$ *has the highest D-classification ability when the condition is met*

(3) $$S_{g}\left(∆P_{j_{i}}\right)=\underset{k}{\max}S_{g}\left(∆P_{k_{i}}\right),$$

*where* $S_{g}\left(∆P_{k_{i}}\right)$ *is the geometric mean in the set of parameter increments* $P_{k}$.

The classification ability of a parameter increases when the geometric mean of its increments tends to the arithmetic mean. Using the geometric mean as an indicator of classification ability excludes the occurrence of zero increments because it brings it to 0. In such a situation, you can replace the zero increments of the $P_{k}$ parameter with a very low value ε such that it does not significantly affect the geometric mean value [29].

**Definition** **3.** *Parameter* $P_{j}$ *has the highest A-classification ability when the condition is met*

(4) $$S_{h}\left(∆P_{j_{i}}\right)=\underset{k}{\max}S_{h}\left(∆P_{k_{i}}\right),$$

*where* $S_{h}\left(∆P_{k_{i}}\right)$ *means the harmonic mean of the parameter increments* $P_{k}$.

The parameter classification ability increases when the harmonic mean of its increments increases to the arithmetic mean of the increments. The harmonic mean is quite resistant to outliers, but its calculation requires that all component values be non-zero. This can be achieved using the method discussed above [29].

**Definition** **4.** *Parameter* $P_{j}$ *has the highest E-classification ability when the condition is met*

(5) $$\underset{i}{\max}\left(∆P_{j_{i}}\right)=\underset{k}{\min}\left(\underset{i}{\max}\left(∆P_{k_{i}}\right)\right).$$

*Therefore, the classification ability assessed using the E-criterion will increase when* $\underset{i}{\max}\left(∆P_{k_{i}}\right)$ *decreases. This coefficient is very poorly resistant to the occurrence of large values. However, its undoubted advantage is simplicity.*

As mentioned earlier, the above classification ability coefficients can be applied after normalizing the parameter values. This distorts information, showing the actual distribution of parameter values. Therefore, it seems more beneficial to use a new coefficient assessing the classification ability of parameters, which does not require data normalization, because its very construction causes it to take values from the range [0, 1]. Let us therefore adopt the following definition.

**Definition** **5.** *Parameter* $P_{j}$ *has the highest nD-classification ability when the condition is met*

(6) $$\frac{S_{g}\left(∆P_{j_{i}}\right)}{S_{a}\left(∆P_{j_{i}}\right)}=\underset{k}{\max}\frac{S_{g}\left(∆P_{k_{i}}\right)}{S_{a}\left(∆P_{k_{i}}\right)},$$

*where* $S_{g}\left(∆P_{k_{i}}\right)$ *and* $S_{a}\left(∆P_{k_{i}}\right)$ *are the geometric mean and arithmetic mean of the increments of the parameter* $P_{k}$ *values, respectively.*

From this definition, it follows that the nD-classification ability of the parameter $P_{k}$ increases when the value of the quotient $\frac{S_{g}\left(∆P_{k_{i}}\right)}{S_{a}\left(∆P_{k_{i}}\right)}$ increases to 1. This coefficient takes values from the range [0,1]. The value of 0 can be achieved when the geometric mean of the increments equals 0, which occurs when at least one of the parameter value increments is zero. The coefficient takes on a maximum value of 1 when the arithmetic mean equals the geometric mean, i.e., when all increments are equal. Therefore, it is a normalized coefficient, even though the values of the analyzed parameters themselves may be non-normalized. This feature of the new coefficient allows for the comparison of non-normalized parameters with any range of values. Using the previous classification ability indices, this was impossible and needed parameter normalization.

### 2.2. Construction of a Complementary Parameter Set with High Classification Capacity for Surface Assessment

In works [29,30,31], the variability of the above-defined classification ability coefficients and the existence and strength of dependencies between them were analyzed. In the article [32], the correlation and regression analysis between the summary classification ability indices and the indices based on selected criteria was presented. Also, the correlation between the parameters with the highest classification ability and the summary indices is examined.

Klichova et al. [33] presented a quantitative comparison of the profile roughness values derived using the new and old ISO standards, along with improvements in the measurement methods for calculating the profile roughness parameters under the new and old ISO standards. Using both the old and new ISO standards, a surface produced by an abrasive water jet served as an example surface for quantitatively comparing profile surface roughness metrics.

This article aims to extend the analysis of the parameters’ correlation with the highest classification ability, published previously [34]. The authors also intend to carry out a differential assessment of the tested technical surfaces using the proposed complementary set of parameters with the highest classification ability.

Data containing values of 83 parameters for 22 surfaces were analyzed. All considered surfaces, although they were created in different machining processes and differ significantly in stereometric features, have approximately the same *St* parameter value. Among the 22 surfaces, there were 7 surfaces after abrasive machining, 7 after erosive machining, 3 abrasive-smoothed surfaces, 2 with high porosity, 2 after operational wear, and 1 with regular topography.

Papers [28,29] presented the full description of the parameters considered in the study. In this work, the parameters described in Table 1 are analyzed. The last three items of the table are new parameters proposed in this work.

To shorten the notation, we will denote the parameter σ(sqrt(Pw)/sqrt(Pw) by σ.

The purpose of creating a complementary set of parameters with high classification ability is to effectively differentiate surfaces that are characterized by differing significance. The steps for making such a set should be as follows:

1. Selection of a set of surfaces that differ significantly in surface structure concerning as many features as possible (e.g., high peaks, deep valleys, large height gradients).
2. Determining the values of the parameters to be tested for selected surfaces.
3. Sorting ascending values of each parameter for all surfaces and calculating the increments of the parameter values.
4. Calculation of the values of the classification ability indices proposed in the work for each parameter (considering the correction of zero increments with very low values for indices (3) and (4)).
5. Selecting several parameters with the highest classification ability for individual criteria from (2) to (6). It is most beneficial to select parameters with high classification ability for several or all criteria. The final number of selected parameters for surface classification should not be too many. For perceptual reasons, there should not be significantly more than 5.
6. Analyzing the correlation between selected parameters and removing the most correlated ones. In the case of removing parameters from the set, supplementing it with the next in the ranking parameters with high classification ability, and reexamining the correlation until obtaining a complementary set of uncorrelated parameters with high classification ability of the required size.
7. Based on the selected parameters, the tested surfaces are differentiated.

**Figure 2:**
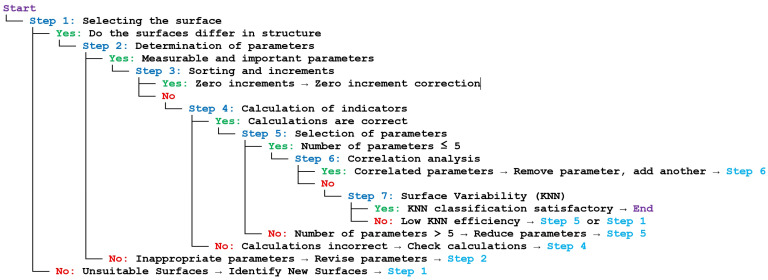
Sample decision tree of a classification model.

This procedure was illustrated in the form of an exemplary decision tree chart. To enhance the decision tree process for creating a complementary set of parameters with high classification capability, the K-Nearest Neighbors (KNN) classification model can be introduced as a method to perform the final differentiation of surfaces (Step 7 of the procedure above). The decision tree incorporating KNN in Step 7 was presented as a text-based graphical representation (Figure 2). KNN leverages the high classification capability of selected parameters to distinguish surfaces more effectively.

## 3. Methods and Basic Analysis

**Figure 3:**
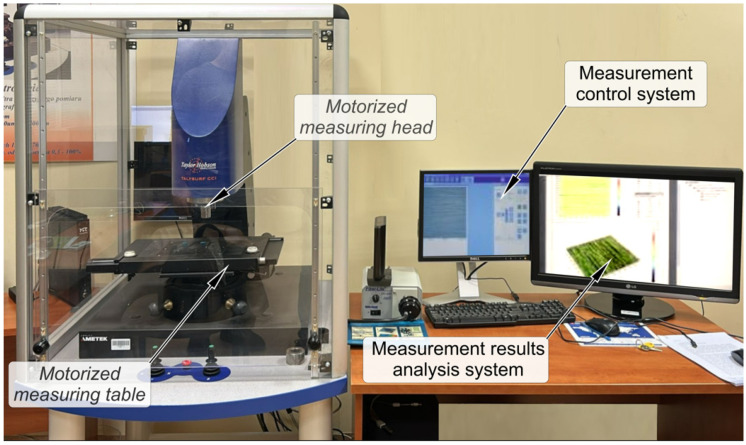
General view of the test stand: Talysurf CCI 6000 profilometer.

The Talysurf CCI 6000 (Taylor Hobson, Leicester, UK) profilometer was used to measure the surface topography (Figure 3) under 20× magnification. This allows for the analysis of surfaces on an elementary area of 0.9 mm × 0.9 mm dimensions. A 1024 × 1024 CCD sensor was used to record data, enabling surface measurement with a horizontal resolution of 0.88 μm. The vertical profilometer resolution is up to 10 pm. Surface analysis was performed using the TalyMap,ver. 9^®^ surface analysis system. The roughness parameter values were determined by ISO 25178-2 [35]

The analysis of the dependence between the parameters S5p, Sp, Sv, Sa, and Vm, which obtained the highest total classification ability, was presented in [30], allowed us to propose the parameters Sa, Sp/Sv, S5p, and Vm as a complementary set used to effectively distinguish surfaces.

The new parameter Sp/Sv was used instead of Sv itself because all the analyzed surfaces were characterized by the value $S_{t}=1$. Therefore, there is a relationship Sp + Sv = 1, which means that these parameters are deterministically related, and their simultaneous use does not make sense. Moreover, the parameter Sp/Sv provides additional information about the tested surface.

**Figure 4:**
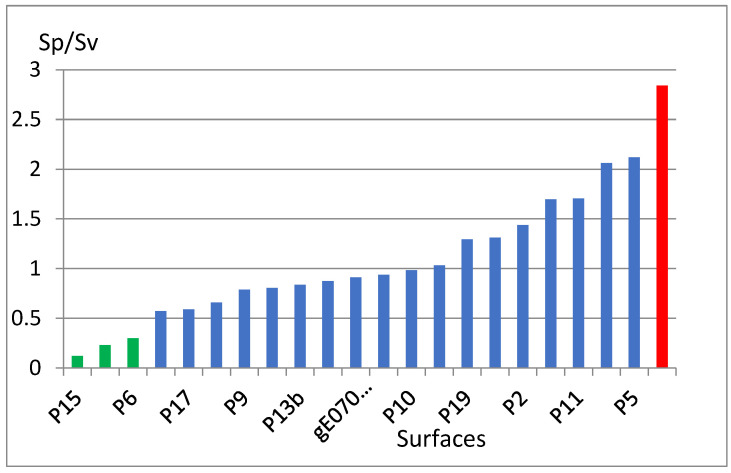
Values of the Sp/Sv parameter for the analyzed surfaces.

For surfaces with a low Sp/Sv coefficient, the ratio of maximum elevation to maximum depth is small. These are the surfaces with the most favorable properties from an operational point of view, where maximum surface smoothness is significantly important. The best surfaces in this respect include P3, P6, and P15 (Figures 42, 45, and 54, respectively). On the other hand, the highest value of maximum elevations to maximum depths is found in surface P16 (Figure 55). The graph of the Sp/Sv parameter values for individual surfaces [30] is shown in Figure 4.

**Figure 5:**
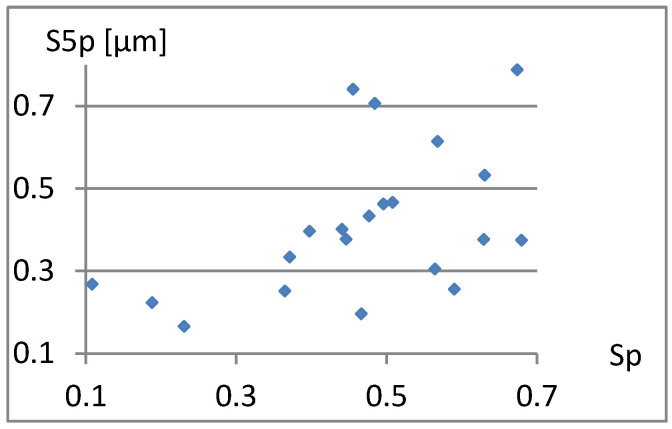
Correlation between Sp and S5p.

**Figure 6:**
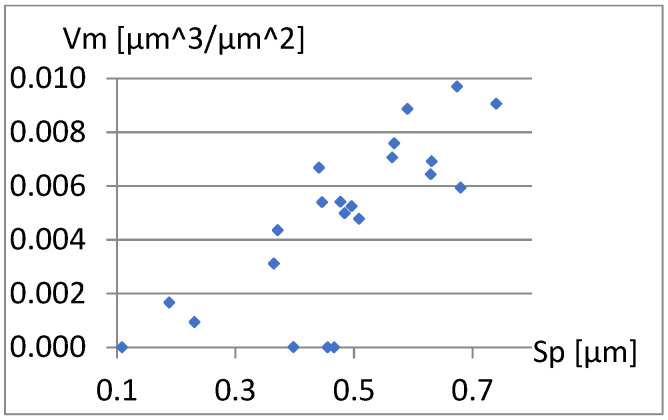
Correlation between Sp and Vm.

The complementary set of parameters does not include the parameter Sp itself, and it is quite strongly correlated with S5p and Vm. These dependencies are visible in Figure 5 and Figure 6.

**Figure 7:**
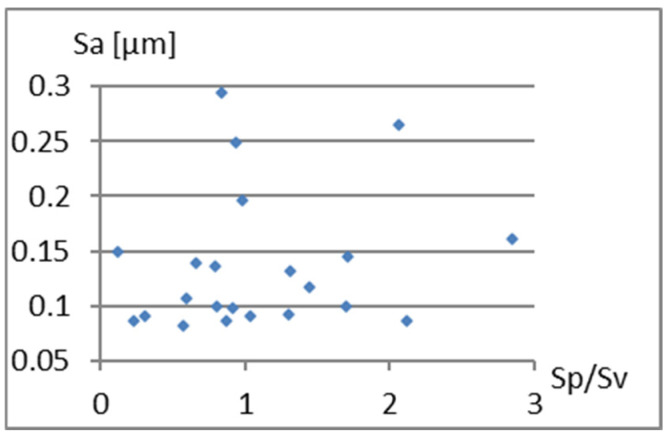
Correlation between Sp/Sv and Sa.

**Figure 8:**
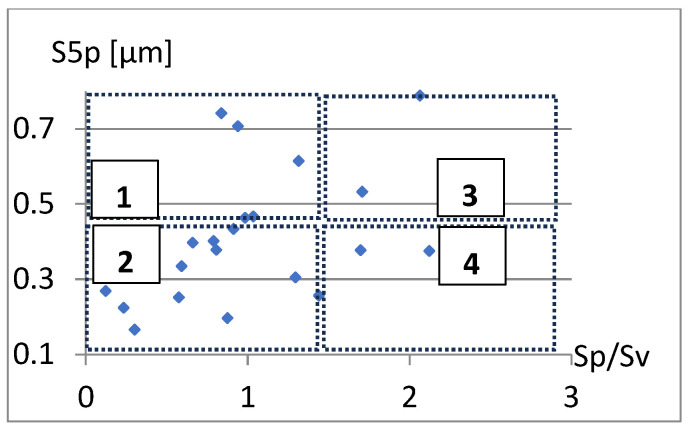
Correlation between Sp/Sv and S5p.

**Figure 9:**
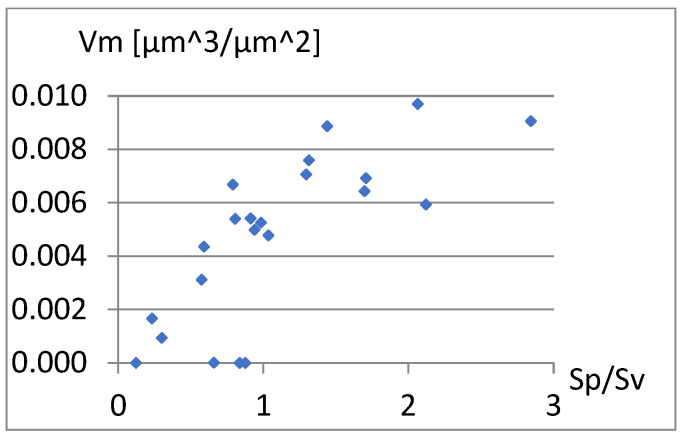
Correlation between Sp/Sv and Vm.

Correlation analysis of the parameters Sa, S5p, and Vm from the parameter Sp/Sv is presented in Figure 7, Figure 8 and Figure 9. Fields 1, 2, 3, and 4 in Figure 8 illustrate: 1—area of significant unevenness with deep fissure-like valleys, 2—area of minor irregularities with significant slender valleys, 3—area of high slender hills, 4—area of moderate hills with a significant gradient.

Based on the graphs presented in Figure 8 and Figure 9, it can be concluded that there is a certain relationship between Sp/Sv and the S5p parameters, and especially Vm. On the other hand, it can be seen (Figure 7, Figure 8 and Figure 9) that for the Sp/Sv parameter close to 1, the remaining parameters obtain values from almost the entire range of values obtained in the study. This would show the lack of dependence between these parameters. Including the Sp/Sv parameter in the complementary set is questionable. It may only depend on the specificity and needs of the surface analysis being conducted.

In addition to the new parameter Sp/Sv proposed in [30], it is also worth considering the parameters S5p/Sv and Vm/S5p. The first one has a similar interpretation to Sp/Sv, but only the height of the five highest peaks is considered in the numerator. The second parameter describes the ratio of the volume of material at a given level per unit area to the height of the five highest peaks on this surface. Both proposed parameters are based on the values of the parameters with high classification ability, as discussed earlier.

**Figure 10:**
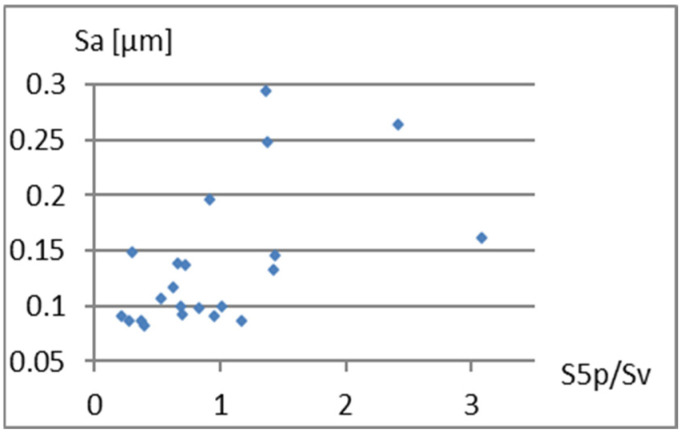
Correlation between S5p/Sv and Sa.

**Figure 11:**
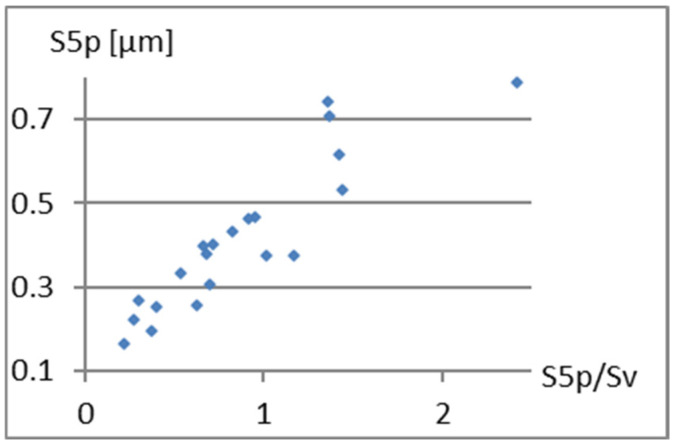
Correlation between S5p/Sv and S5p.

**Figure 12:**
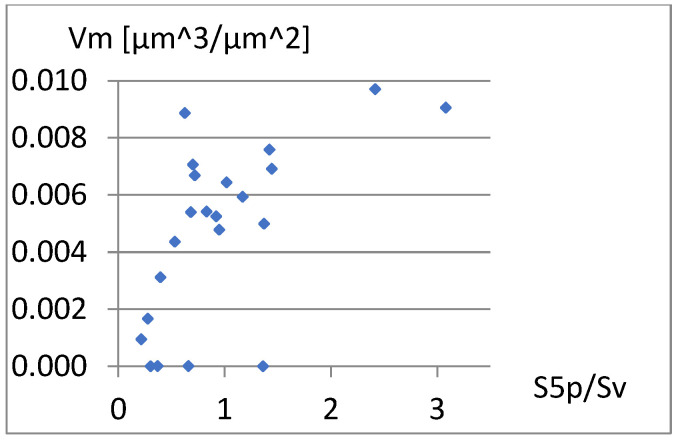
Correlation between S5p/Sv and Vm.

To assess the possibility of including these parameters in a complementary set of parameters with high classification ability, their correlation with the previously adopted elements of this set, i.e., Sa, S5p, and Vm, will be examined. Figure 10, Figure 11 and Figure 12 present correlation diagrams of the dependencies between S5p/Sv and these parameters.

Analysis of the above graphs confirms a strong correlation between S5p/Sv and S5p, which is expected under its mathematical nature. The linear correlation coefficient between these parameters is r=0.89. The correlation from Vm is also important. Although this relationship is rather nonlinear, even the linear correlation coefficient is r=0.61. Therefore, S5p/Sv should not be added to the complementary set constructed so far. The above parameter can be used instead of the S5p parameter.

**Figure 13:**
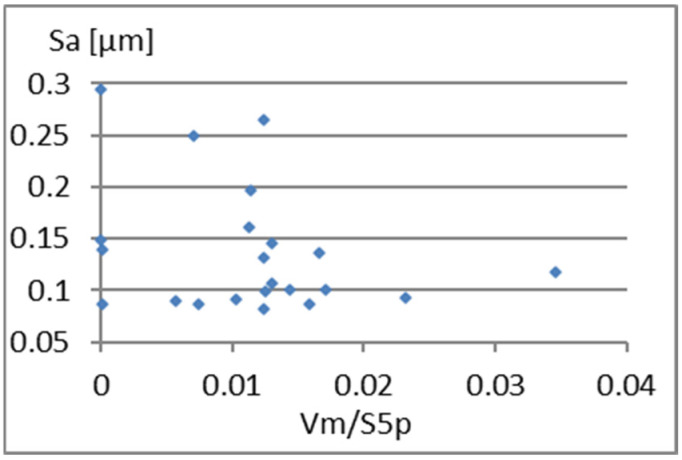
Correlation between Vm/S5p and Sa.

**Figure 14:**
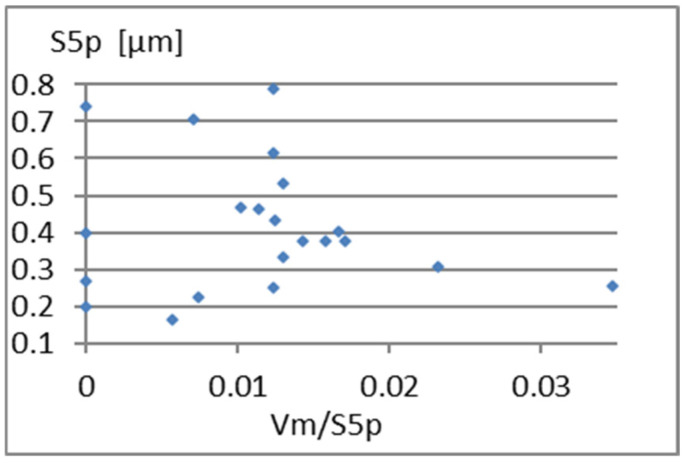
Correlation between Vm/S5p and S5p.

**Figure 15:**
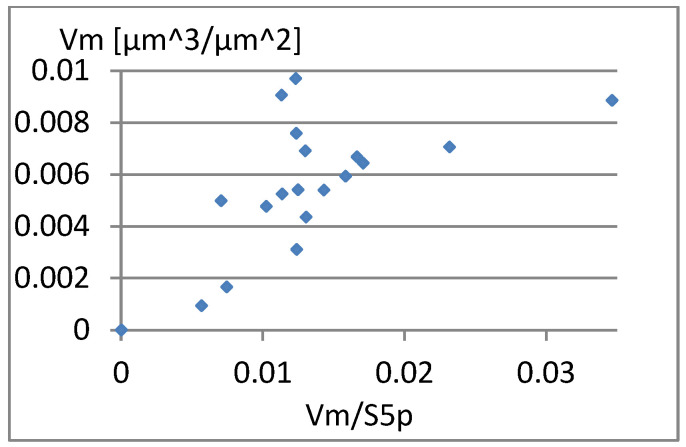
Correlation between Vm/S5p and Vm.

Another interesting parameter is Vm/S5p. Graphs showing its correlation with elements of the complementary set of parameters considered are presented in Figure 13, Figure 14 and Figure 15. A small correlation between this parameter and S5p (Figure 14) is caused by the different shape of the unevenness and the possibility of surface elevations in the form of peaks with an unfavorably large gradient. A significant correlation (linear correlation coefficient equals *r* = 0.77) of Vm/S5p with Vm is shown in Figure 15. Considering Vm/S5p as an element of the complementary set of parameters is possible only in the case of its exchange with Vm.

Due to the small number of parameters included in the constructed complementary set of parameters used to differentiate surfaces, it is worth considering its extension with additional elements. Other parameters with high total classification ability are Sq, S10z, σ, and Vmp [30].

**Figure 16:**
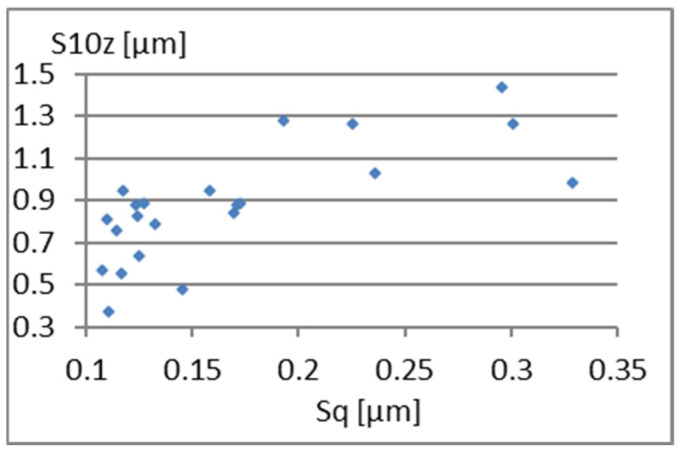
Correlation between Sq and S10z.

**Figure 17:**
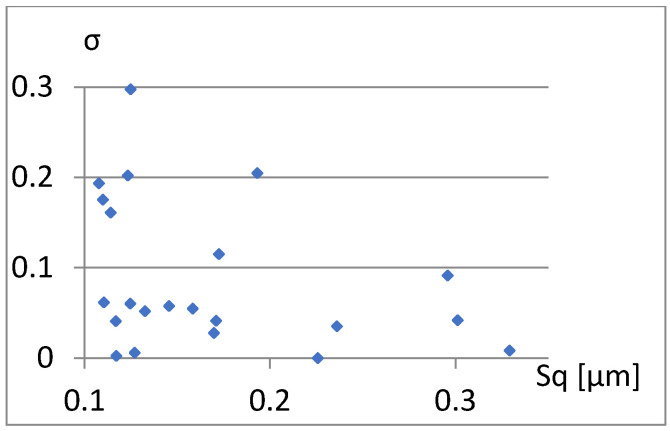
Correlation between Sq and σ.

**Figure 18:**
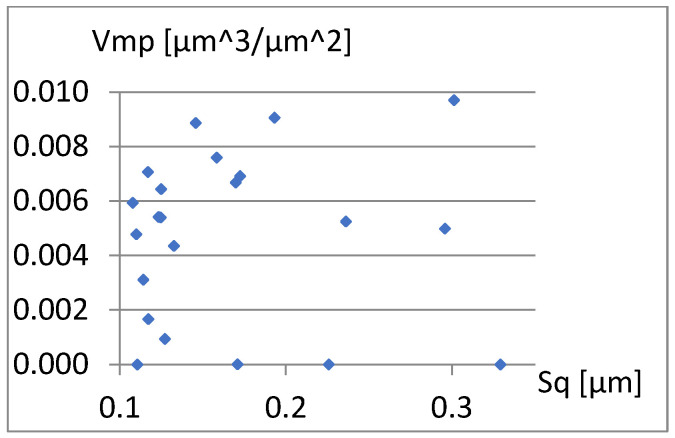
Correlation between Sq and Vmp.

**Figure 19:**
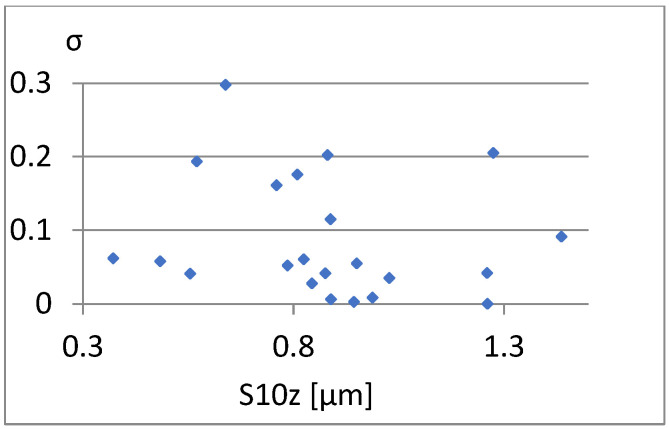
Correlation between S10z and σ.

**Figure 20:**
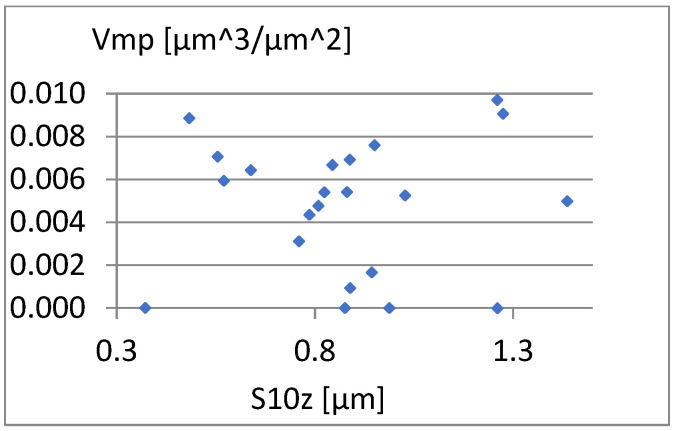
Correlation between S10z and Vmp.

**Figure 21:**
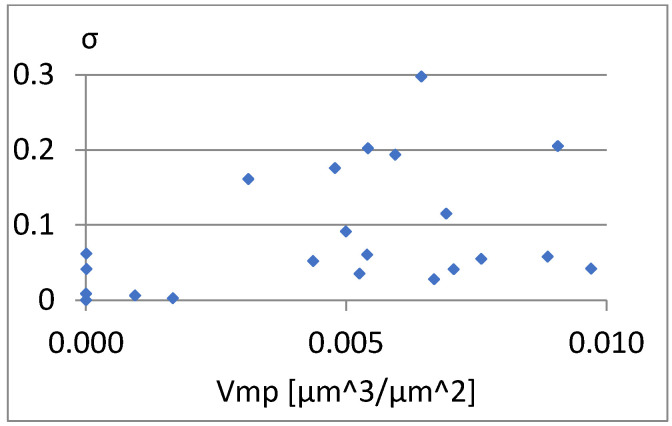
Correlation between Vmp and σ.

**Figure 22:**
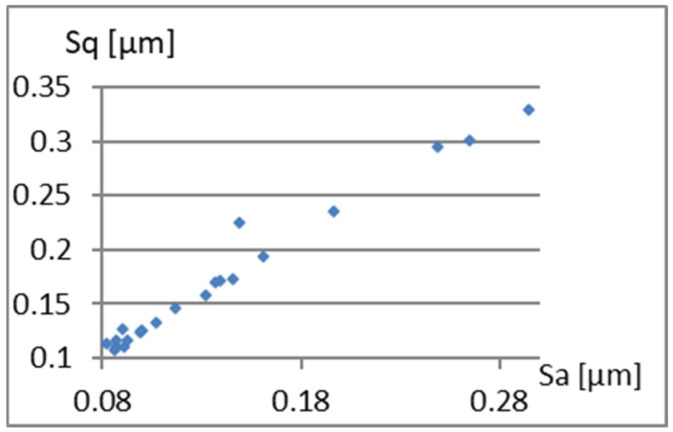
Correlation between Sa and Sq.

We will now assess the existence of dependencies between these parameters (Figure 16, Figure 17, Figure 18, Figure 19, Figure 20 and Figure 21). Figure 16 shows a strong dependency between Sq and S10z. The linear correlation coefficient is r=0.73. The remaining dependencies are of little importance. Due to the Sq factor’s strong dependency on the Sa (Figure 22), this parameter will not be considered in further analysis. The linear correlation coefficient for the parameters Sq and Sa is almost 0.99.

**Figure 23:**
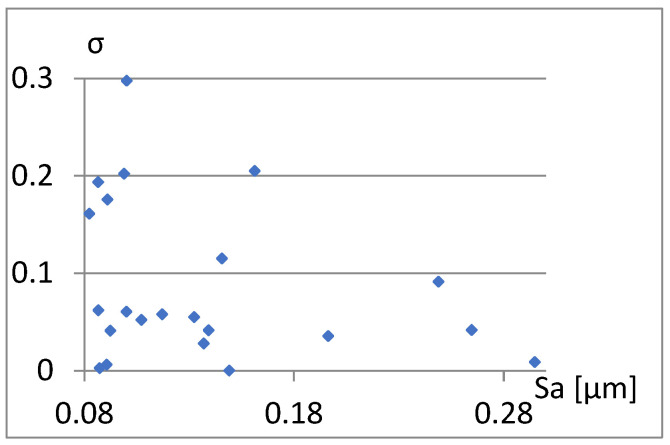
Correlation between Sa and σ.

**Figure 24:**
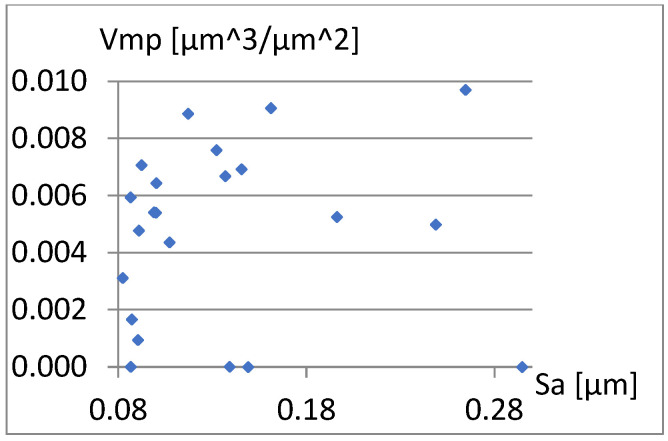
Correlation between Sa and Vmp.

**Figure 25:**
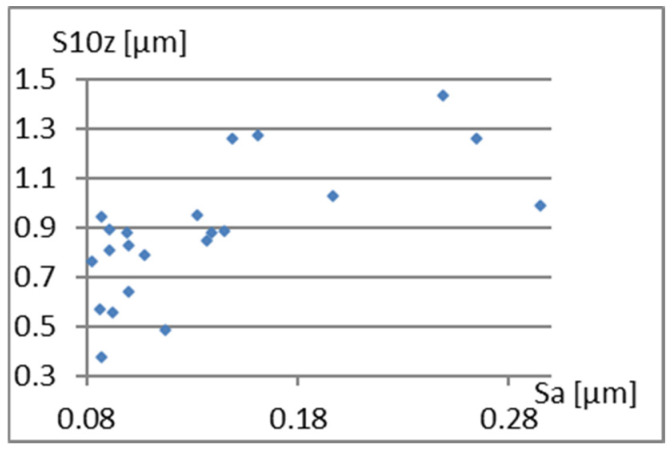
Correlation between Sp/Sv and S10z.

Therefore, it remains to examine the correlation between the parameters already included in the complementary set being created and S10z, σ, and Vmp. Figure 23, Figure 24 and Figure 25 show that the values of these parameters are not dependent on Sa.

**Figure 26:**
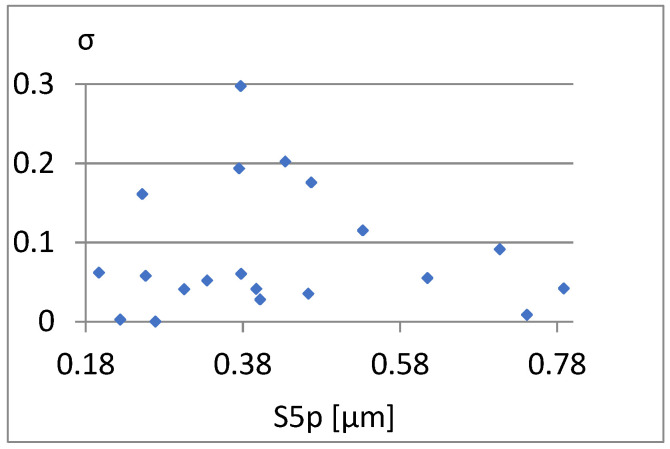
Correlation between S5p/Sv and σ.

**Figure 27:**
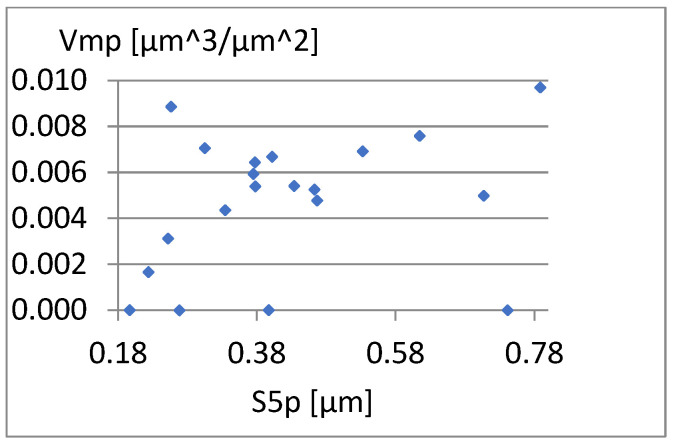
Correlation between S5p and Vmp.

**Figure 28:**
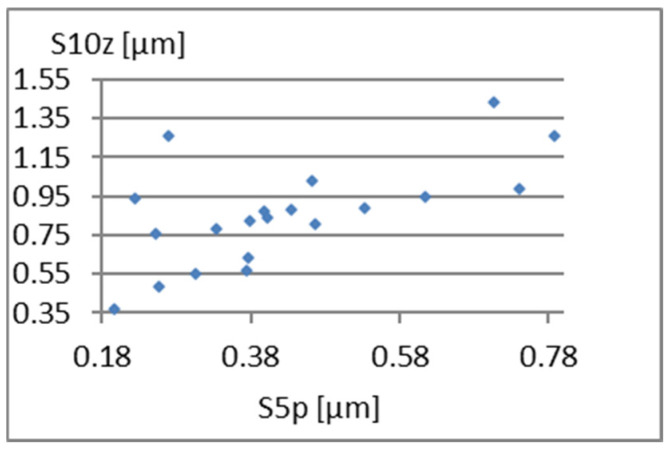
Correlation between S5p and S10z.

Figure 26, Figure 27 and Figure 28 present the dependencies of σ, Vmp, and S10z on the S5p parameter. Only the last one seems to be more important. For S10z values close to 0.95, the S5p parameter assumes values from almost its entire range in this research. This indicates a small correlation between these parameters.

**Figure 29:**
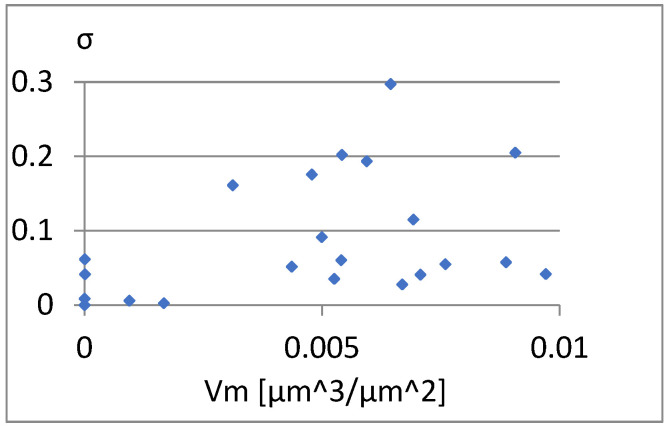
Correlation between Vm and σ.

**Figure 30:**
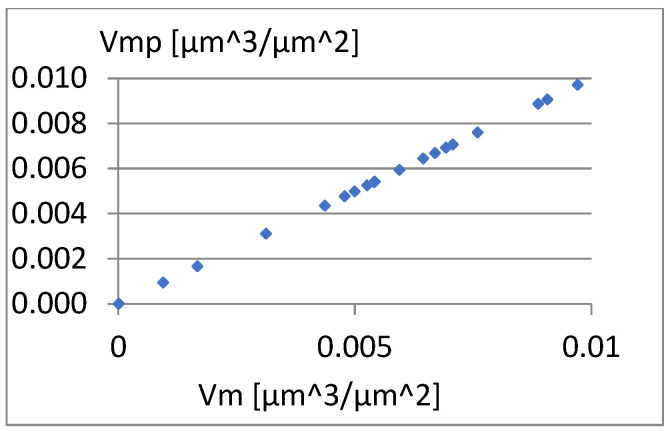
Correlation between Vm and Vmp.

**Figure 31:**
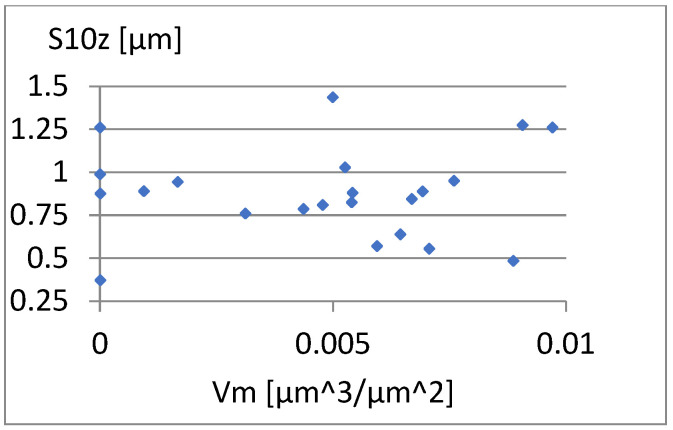
Correlation between Vm and S10z.

Figure 29, Figure 30 and Figure 31 present the correlation between Vm and the parameters σ, Vmp, and S10z. Figure 30 shows a strong linear relationship between the parameters Vm and Vmp. The linear correlation coefficient between them is approximately 1. However, based on the diagrams in Figure 29 and Figure 31, it can be assessed that there is no significant relationship between S10z, σ, and the parameter Vm.

Considering the above analyses, we obtain an exemplary complementary set of the following parameters with high classification ability: Sa, S5p, Vm, S10z, and σ.

**Figure 32:**
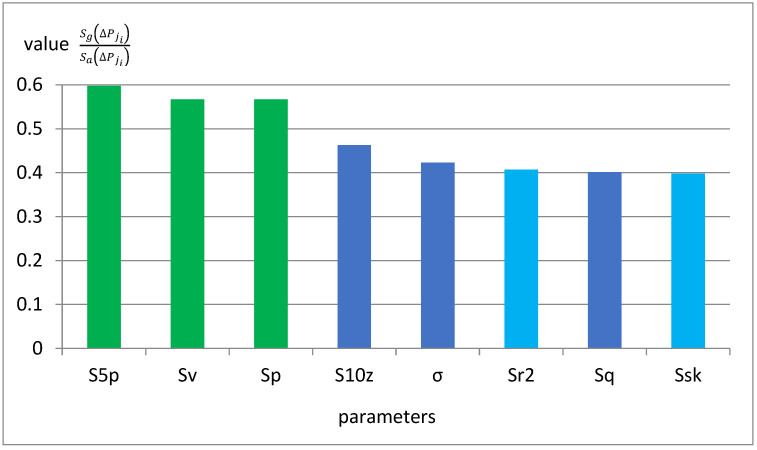
Example indicator for 8 parameters with the highest classification ability.

In Section 2.1, a new classification ability coefficient was proposed, which can be applied to non-standardized parameter values. Figure 32 presents the values of this indicator for 8 parameters with the highest classification ability.

The ninth parameter has a significantly lower coefficient value. It is worth noting that the highest classification ability of the parameters S5p, Sv, and Sp is also repeated for this criterion. Among the parameters with high classification ability to the new criterion, presented in Figure 32, only the Sr2 and Ssk parameters were not considered.

**Figure 33:**
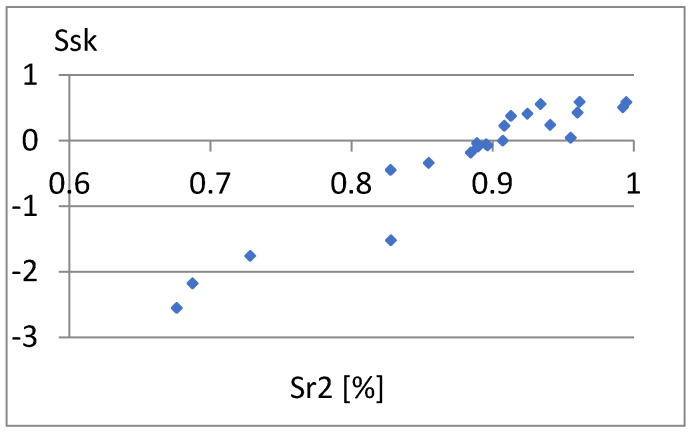
Correlation between Sr2 and Ssk.

**Figure 34:**
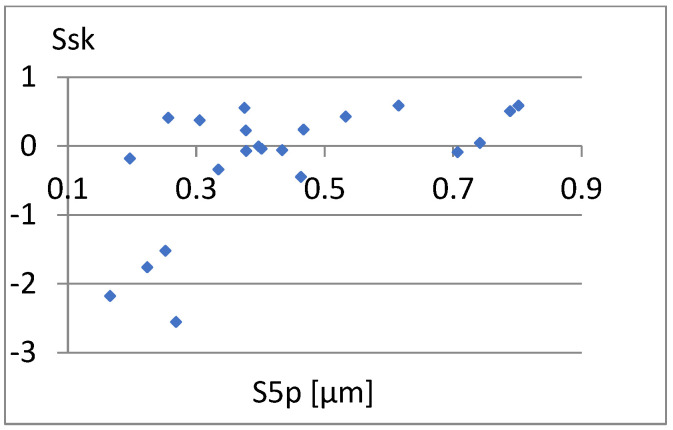
Correlation between S5p and Ssk.

**Figure 35:**
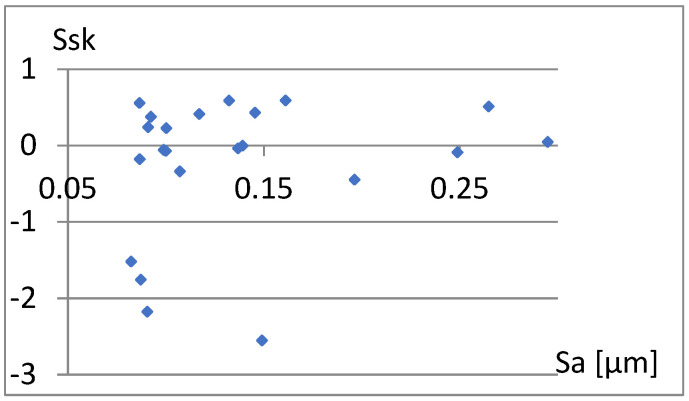
Correlation between Sa and Ssk.

**Figure 36:**
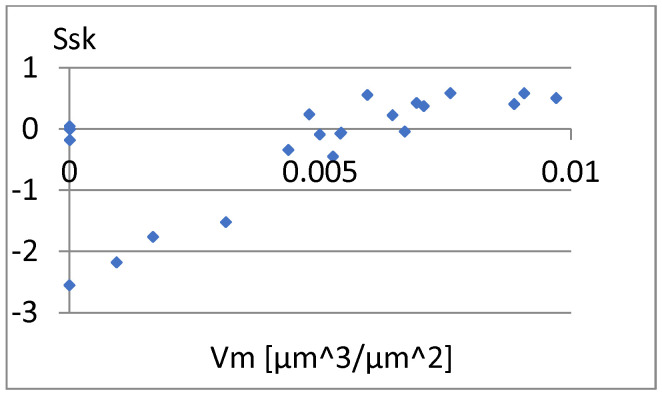
Correlation between Vm and Ssk.

**Figure 37:**
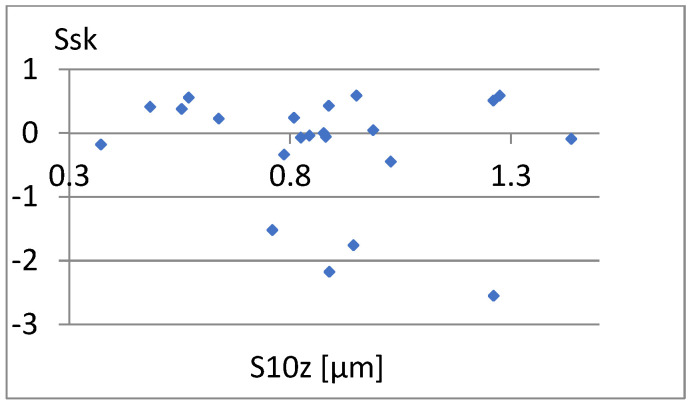
Correlation between S10z and Ssk.

**Figure 38:**
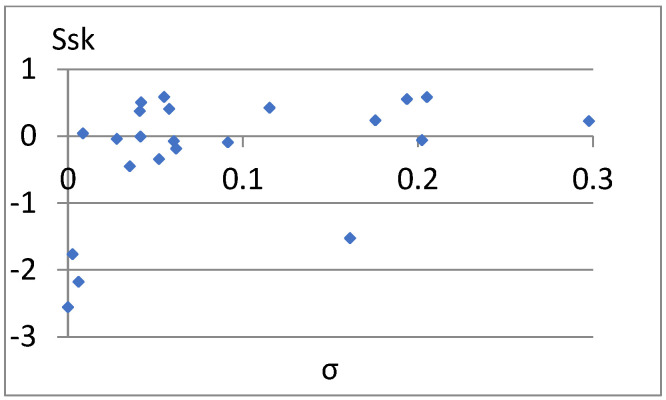
Correlation between σ and Ssk.

Figure 33 shows a relatively strong correlation between these parameters. The linear correlation coefficient between them is r = 0.96. It is enough to add one of them to the previously proposed set of parameters for differentiating surfaces. Due to the different nature of the Ssk factor of the previous set of parameters, it seems appropriate to choose this parameter. Now, let us examine the correlation between it and the remaining parameters of the constructed set (Figure 34, Figure 35, Figure 36, Figure 37 and Figure 38).

Figure 36 shows a significant dependence between Ssk and Vm. This is confirmed by the value of the linear correlation coefficient between them, which is r=0.67. Therefore, including Ssk in the constructed complementary set of parameters with high classification ability is doubtful. Its inclusion may be supported only by the different nature of this parameter. It may be important considering its considerable independence from other parameters. Including the parameter Ssk in the search set may be important because it describes a different aspect of the surface than the other parameters.

**Figure 39:**
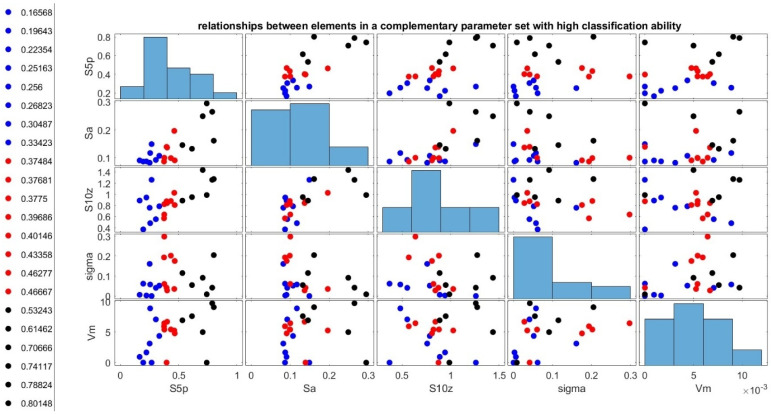
Values of the new classification ability coefficient for the parameters with the highest classification ability.

Therefore, a complementary set of parameters with high classification ability, which can be proposed based on the above analyses, is the set of parameters S5p, Sa, S10z, σ, and Vm. The visualization of the relationships between them and histograms of their values obtained in the study are presented in Figure 39. The parameter σ is described there as the sigma.

## 4. Discriminant Analysis of the Considered Surfaces Created in Different Machining Processes

Section 2.2 presented the construction of an exemplary set of uncorrelated parameters with high summary classification ability. The objective of this set generation is to use it to assess the degree of differentiation of the analyzed surfaces.

Because none of the parameters described in the literature can unequivocally distinguish all significantly different surfaces, there is a need to create a complementary set of parameters that will allow for such an assessment. The criterion of classification ability was assumed to be the degree of compliance of the distribution of parameter values with the uniform distribution for significantly different surfaces. Following this principle, several criteria of classification ability were defined, as well as summary criteria based on them [29,30,31]. It is also important to correlate the parameters used as little as possible to prevent duplicate information when using them.

**Figure 40:**
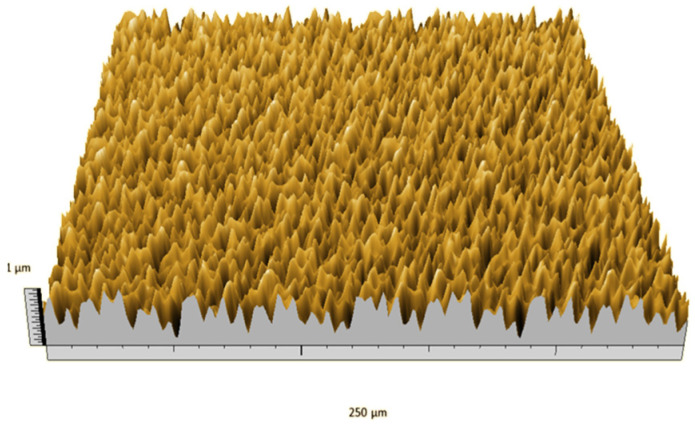
Surface image P1.

**Figure 41:**
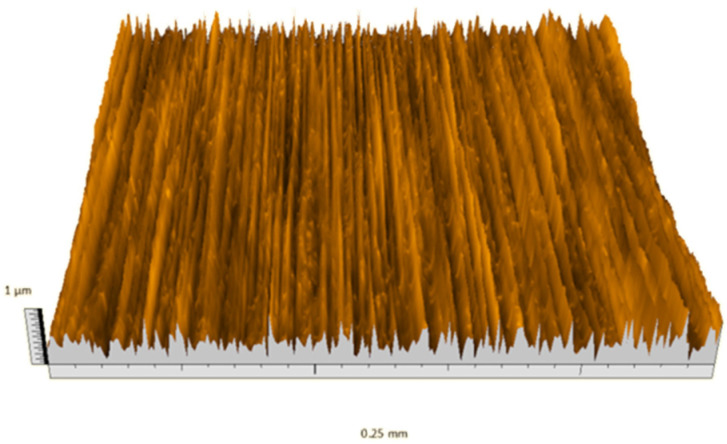
Surface image P2.

**Figure 42:**
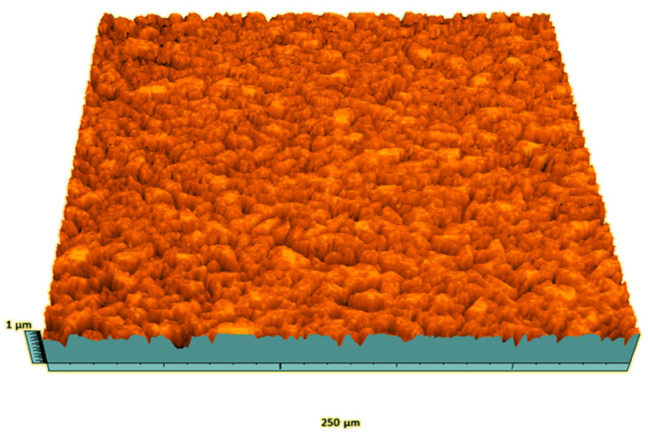
Surface image P3.

**Figure 43:**
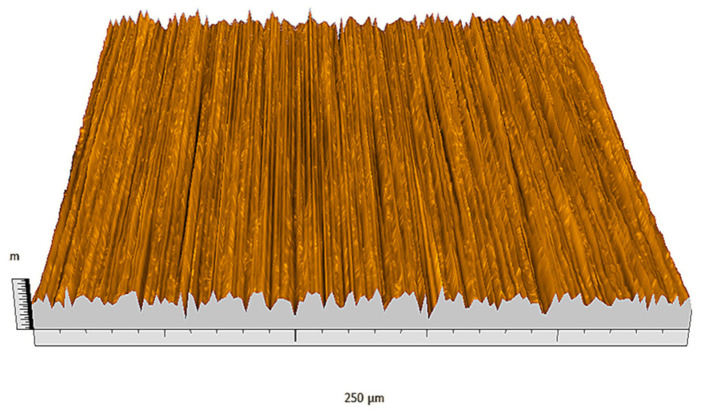
Surface image P4.

**Figure 44:**
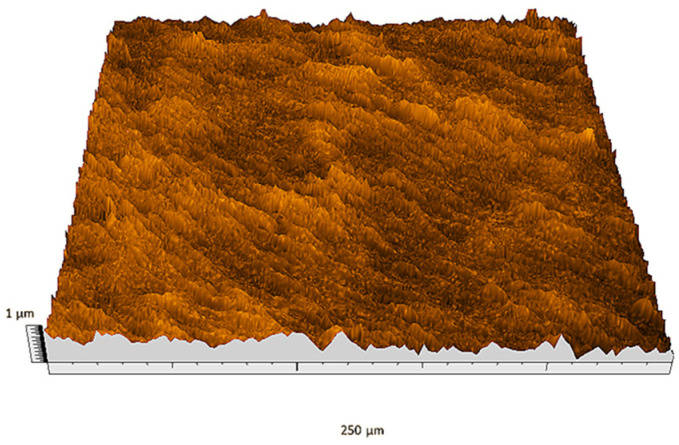
Surface image P5.

**Figure 45:**
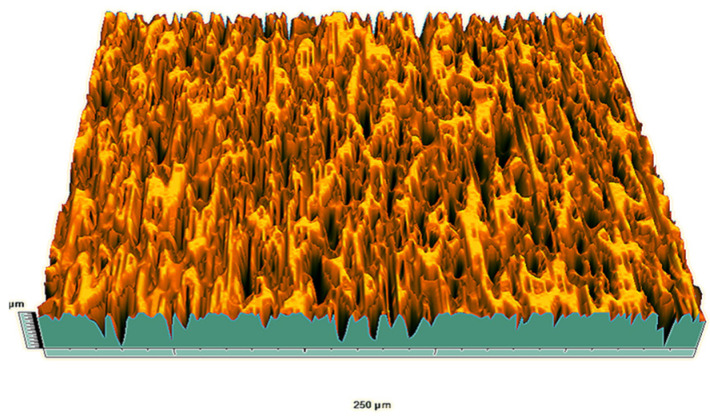
Surface image P6.

**Figure 46:**
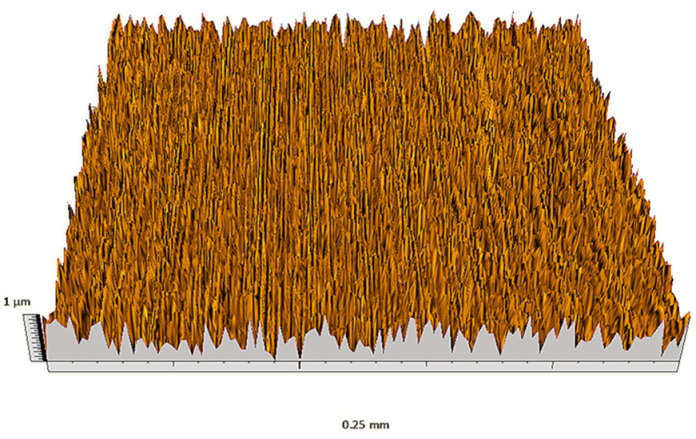
Surface image P7.

**Figure 47:**
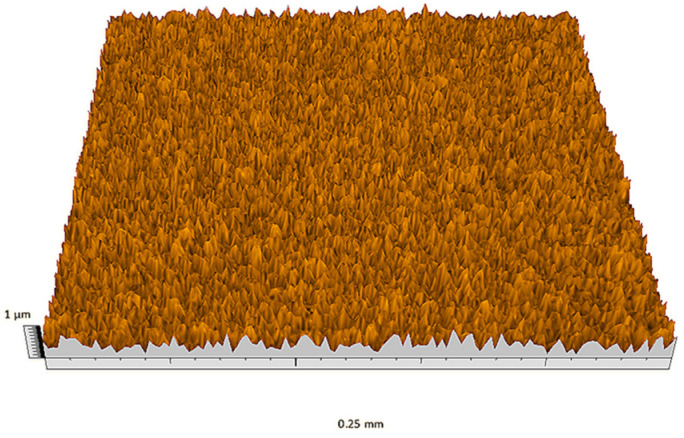
Surface image P8.

**Figure 48:**
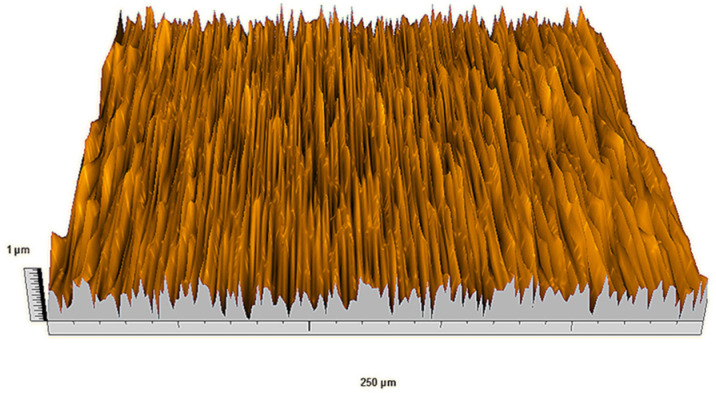
Surface image P9.

**Figure 49:**
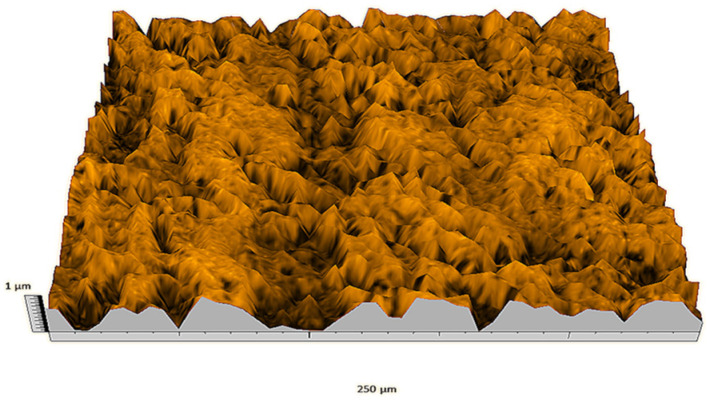
Surface image P10.

**Figure 50:**
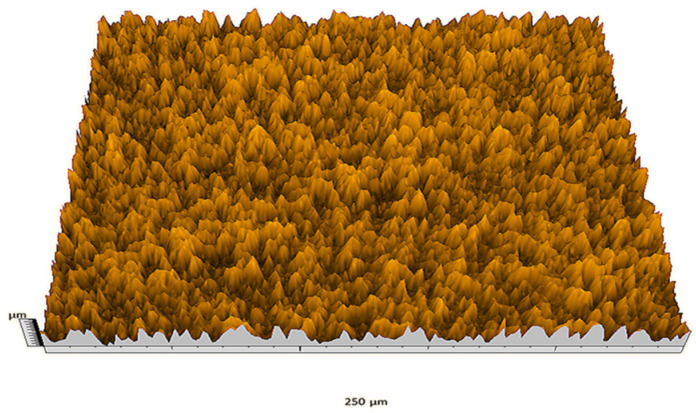
Surface image P11.

**Figure 51:**
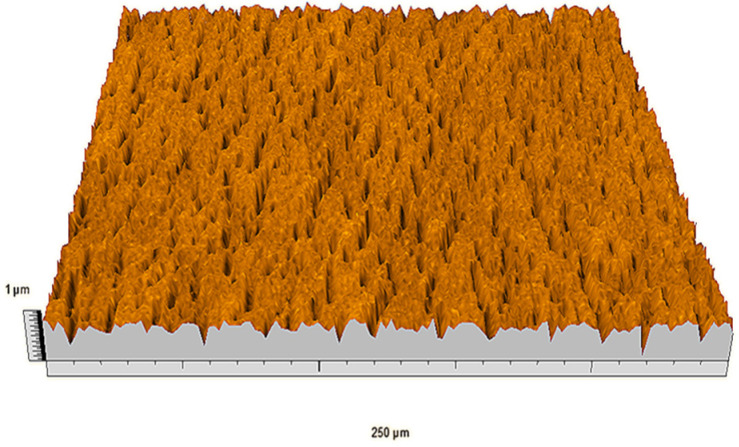
Surface image P12.

**Figure 52:**
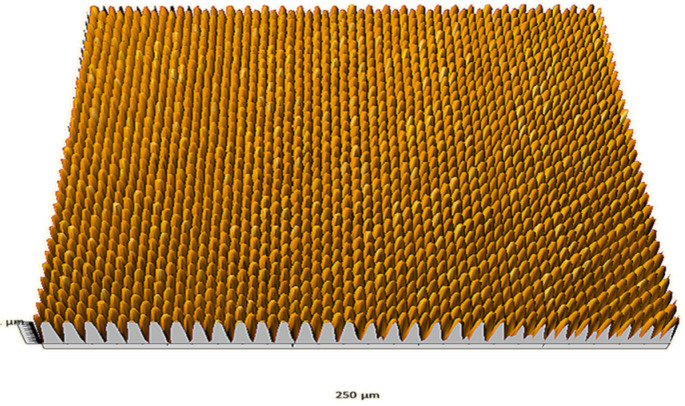
Surface image P13.

**Figure 53:**
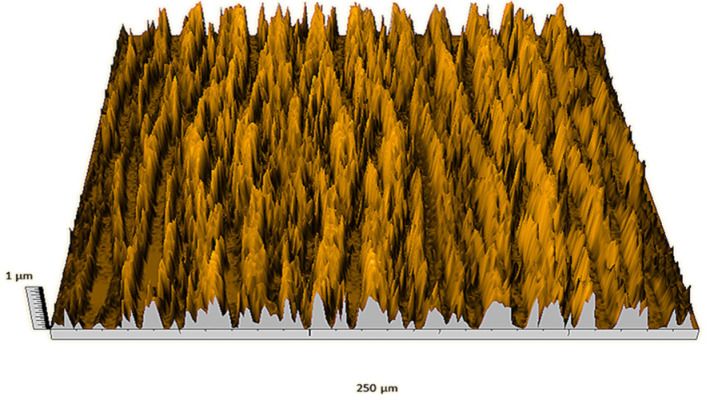
Surface image P14.

**Figure 54:**
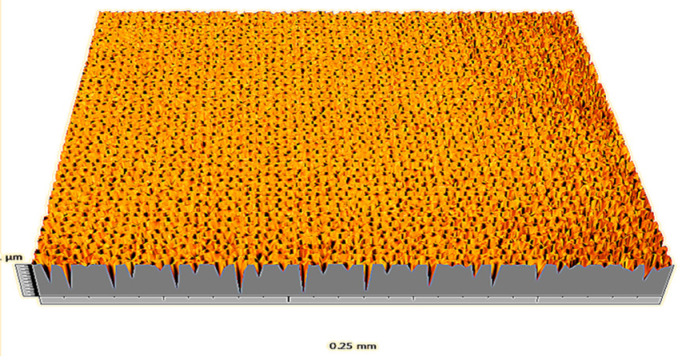
Surface image P15.

**Figure 55:**
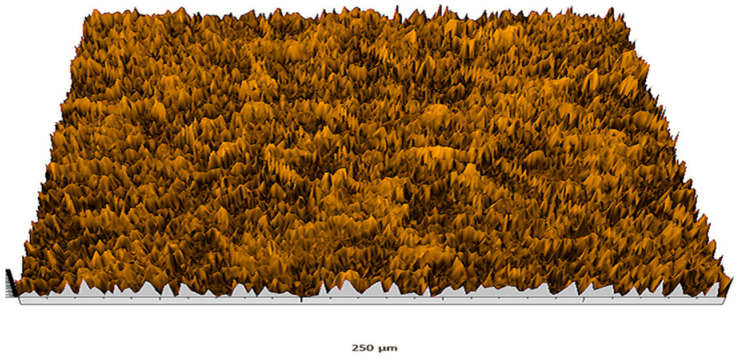
Surface image P16.

**Figure 56:**
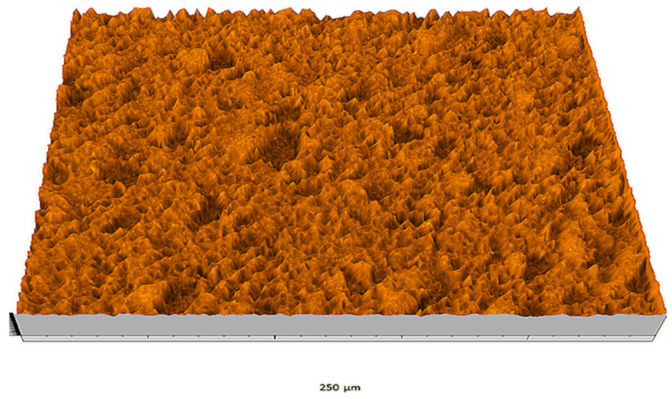
Surface image P17.

**Figure 57:**
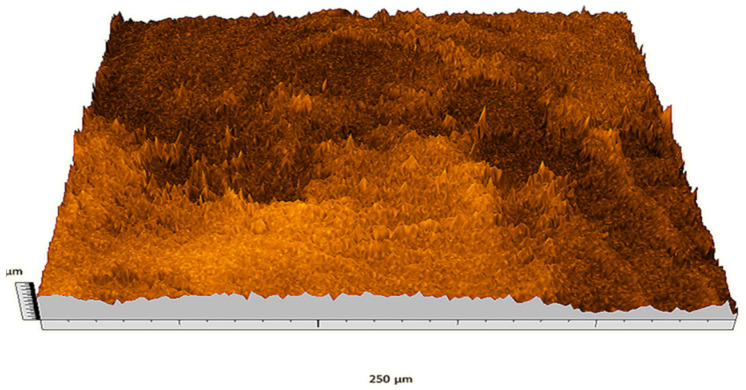
Surface image P18.

**Figure 58:**
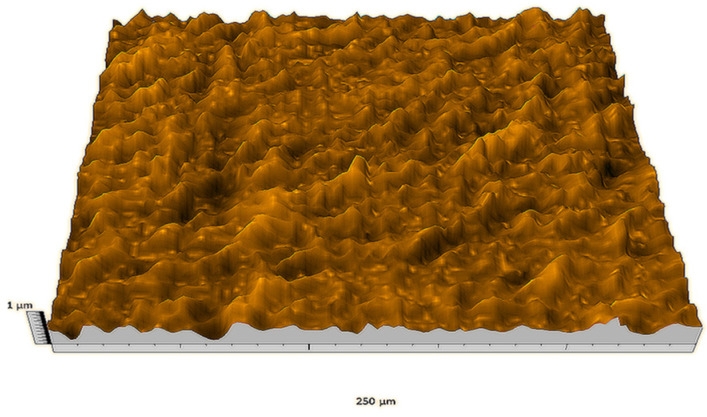
Surface image P19.

**Figure 59:**
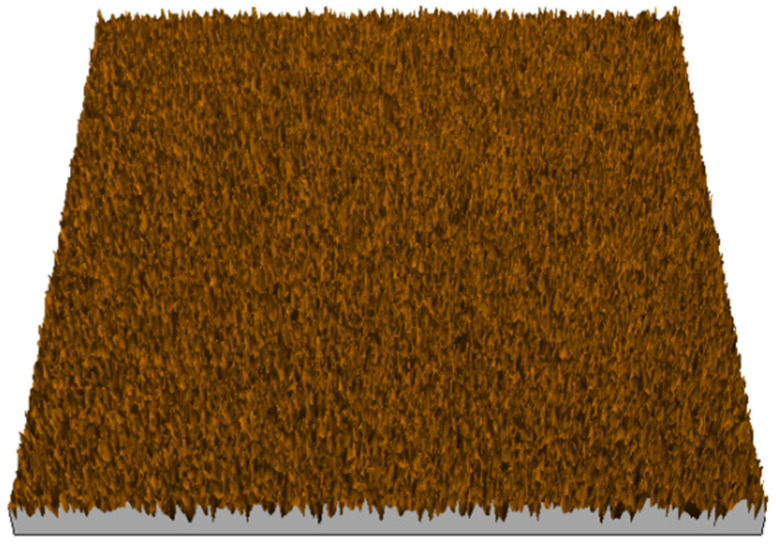
Surface image gE070027.

**Figure 60:**
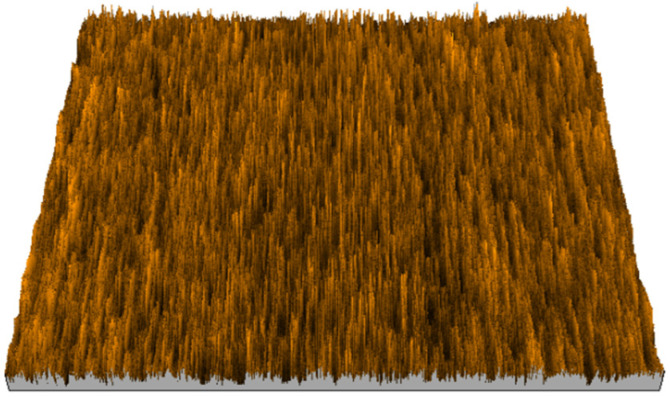
Surface image S0493.

**Figure 61:**
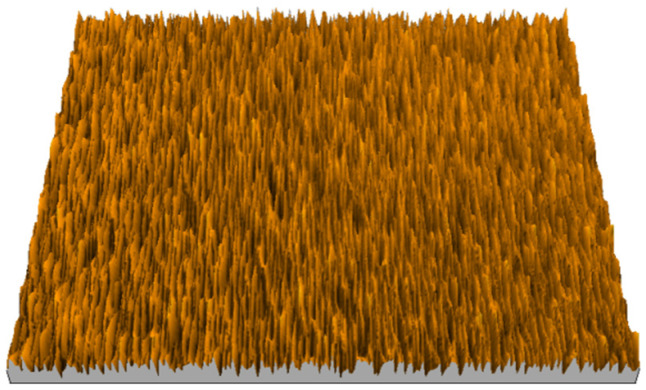
Surface image g030063.

According to the criteria above, the analysis included in the previous chapter allows us to propose Sa, S5p, S10z, Vm, σ, and possibly Ssk as a complementary set of parameters for distinguishing significantly different surfaces. The obtained set of parameters is also diversified in terms of the type of parameters. It contains both height and volume parameters related to the surface areas of the elevations and defining the asymmetry of the surface from the point of view of the parameter Ssk. In this chapter, the analysis of the assessed surfaces concerning the degree of their differentiation is presented. Figure 40, Figure 41, Figure 42, Figure 43, Figure 44, Figure 45, Figure 46, Figure 47, Figure 48, Figure 49, Figure 50, Figure 51, Figure 52, Figure 53, Figure 54, Figure 55, Figure 56, Figure 57, Figure 58, Figure 59, Figure 60 and Figure 61 show exemplary views of 22 analyzed surfaces [28]. The presented surfaces include surfaces after abrasive machining (P2, P4, P7, P9, P14, S0493, g030063), surfaces after erosion machining (P1, P8, P11, P16, P17, P19, gE070027), abrasively smoothed surfaces (P3, P12, P15), highly porous surfaces (P6, P10), a surface with regular topography (P13) and surfaces after operational wear (P5, P18).

**Figure 62:**
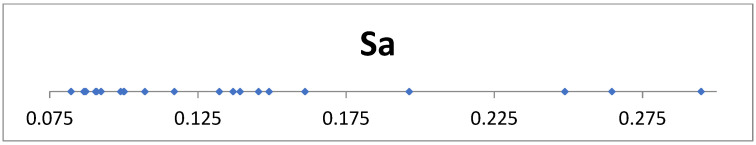
Sa parameter values for individual surfaces.

**Figure 63:**
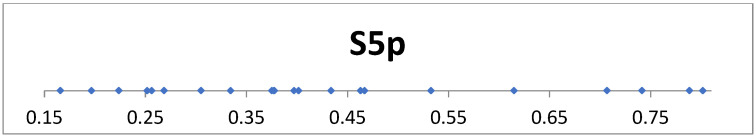
S5p parameter values for individual surfaces.

**Figure 64:**
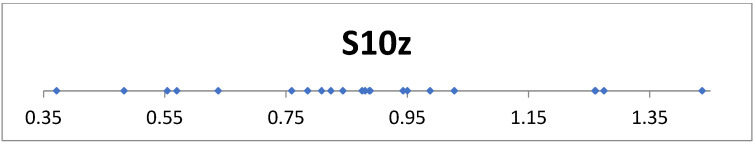
S10z parameter values for individual surfaces.

**Figure 65:**
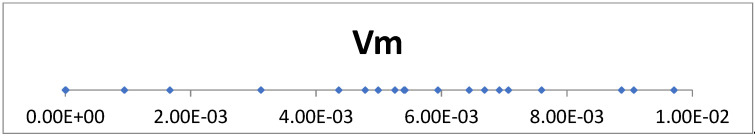
Values of the Vm parameter for individual surfaces.

**Figure 66:**
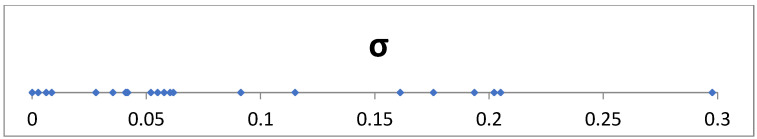
Values of the parameter σ for individual surfaces.

**Figure 67:**
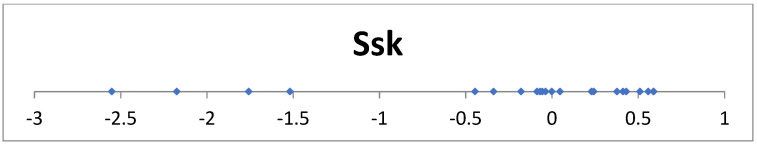
Ssk parameter values for individual surfaces.

Figure 62, Figure 63, Figure 64, Figure 65, Figure 66 and Figure 67 present the values of parameters from the complementary set for individual surfaces. Assessing the degree of differentiation of the analyzed surfaces based on them, it can be seen that the Vm parameter, although it does not have the highest total classification ability in this group, allows for much better differentiation of the assessed surfaces to the remaining parameters (Figure 65). However, the parameter that stands out in terms of the ability to effectively distinguish surfaces is S5p (Figure 63). It also has the highest total classification ability and the highest classification abilities due to the individual criteria described in the first chapter. A comparison of the chart in Figure 67 with the other figures indicates a low classification ability of the Ssk parameter to other parameters from the selected set. Similar conclusions can be drawn when analyzing the Sa parameter (Figure 62).

Based on Figure 63, the following groups of surfaces can be distinguished, which are indistinguishable from the point of view of parameter S5p: (P2, P12, P15), (P5, g030063, S0493), (P1, P9), (P8, P10), (P14, P16). The values of parameter S5p for the remaining surfaces differ significantly, highlighting that this parameter distinguishes them. When assessing the degree of surface differentiation based on the Vm parameter, the following groups of surfaces can be distinguished, which do not differ significantly from the point of view of this parameter (Figure 65): (P1, P4, P13, P15), (P8, P10, g030063, gE070027), (P9, P11, P19, S0493) and (P2, P16). For the remaining surfaces, the values of this parameter differ significantly, and they can be easily distinguished by it.

In the groups of surfaces that cannot be distinguished using the analyzed parameters, only the pair of surfaces P8 and P10 cannot be distinguished by both parameters. The first surface was created as the erosion processing result, while the second is a highly porous surface (as can be seen in Figure 47 and Figure 49); they are visually different from each other. This pair can be effectively distinguished by the parameter Sa.

## 5. Conclusions

The new classification ability index allows for omitting the normalization parameter values step when analyzing its classification ability, as necessary when using the classification ability coefficients described earlier in the literature. Omitting the normalization of parameters is beneficial due to the influence of normalization on the distribution of parameter values, which can distort the assessment results of their classification ability.

Based on the analysis, a set of parameters with high classification ability that are not correlated with each other can be constructed, allowing for effective surface differentiation to be considered in the research. The developed complementary set of parameters consists of Sa, S5p, S10z, Vm, and σ. It is also possible to add the Ssk parameter, with a significant classification ability assessed using a new classification ability index. Although it has a much lower position in the summary assessment of classification ability [30], its advantage lies in the differentiation of the analyzed surface feature.

Due to interpretation properties, some parameters from this set can be replaced by other, strongly correlated parameters with high classification ability. An example could be the parameters Sa and Sq. Due to their strong correlation, it should not appear together in a complementary set of parameters with high classification ability. However, if it’s advisable due to the specificity of features described by these parameters, in the searched set of surfaces differentiating parameters, the Sa parameter may be replaced by Sq. Similarly, the parameter S5p can be replaced by the strongly correlated S5p/Sv or correlated Sp/Sv. Similarly, instead of the parameter Vm in the complementary set, one of the strongly correlated parameters with high classification ability, Vmp or S5p/Sv, or Vm/S5p as well as Sp/Sv, can be included.

It should be noted that the proposed set of parameters with high classification capability is closely related to the specific research. However, the methodology proposed in the article is general and allows for the creation of an analogous set, taking into account surface features important for a specific application. The proposed assessment indicators of the classification capability of the parameters are the main elements of this methodology. It constitutes the basis for the complementary set of creation parameters characterized by high classification capability.

## Figures and Tables

**Table 1:** List of parameters included in the analysis (own study based on [29]).

Symbol	Unit	Context	Description
S5p	μm	slash = 5%	height of 5 surface summits
S10z	μm	slash = 5%	height of 10 surface points
Sa	μm		arithmetic mean deviation of the surface
Sp	μm		maximum height of summits
Sq	μm		root-mean-square deviation of the surface
St	μm		total height of the surface
Sv	μm		maximum depth of valleys
Vm	μm^3^/μm^2^	*p* = 10%	material volume at a given depth
Vmp	μm^3^/μm^2^	*p* = 10%	material volume of peaks
σ(sqrt(Pw)/sqrt(Pw)		h = 0.2 St	ratio of the standard deviation of the square roots of the summit areas to the square root of the mean summit area
Sr2	%	Gaussian filter, 0.8 mm	lower material ratio
Ssk			skewness of the height distribution
Sp/Sv			height of 5 surface summits
S5p/Sv			height of 10 surface points
Vm/S5p		*p* = 10%	arithmetic mean deviation of the surface

## Data Availability

The data presented in this study are available on request from the corresponding author due to privacy.
